# Markers of T Cell Senescence in Humans

**DOI:** 10.3390/ijms18081742

**Published:** 2017-08-10

**Authors:** Weili Xu, Anis Larbi

**Affiliations:** 1 Biology of Aging Program and Immunomonitoring Platform, Singapore Immunology Network (SIgN), Agency for Science Technology and Research (A*STAR), Immunos Building, Biopolis, Singapore 138648, Singapore; xu_weili@immunol.a-star.edu.sg; 2 School of Biological Sciences, Nanyang Technological University, Singapore 637551, Singapore; 3 Department of Microbiology, National University of Singapore, Singapore 117597, Singapore; 4 Department of Geriatrics, Faculty of Medicine, University of Sherbrooke, Sherbrooke, QC J1K 2R1, Canada; 5 Faculty of Sciences, University ElManar, Tunis 1068, Tunisia

**Keywords:** human aging, immunosenescence, immune system, T cells, senescence, markers, phenotyping

## Abstract

Many countries are facing the aging of their population, and many more will face a similar obstacle in the near future, which could be a burden to many healthcare systems. Increased susceptibility to infections, cardiovascular and neurodegenerative disease, cancer as well as reduced efficacy of vaccination are important matters for researchers in the field of aging. As older adults show higher prevalence for a variety of diseases, this also implies higher risk of complications, including nosocomial infections, slower recovery and sequels that may reduce the autonomy and overall quality of life of older adults. The age-related effects on the immune system termed as “immunosenescence” can be exemplified by the reported hypo-responsiveness to influenza vaccination of the elderly. T cells, which belong to the adaptive arm of the immune system, have been extensively studied and the knowledge gathered enables a better understanding of how the immune system may be affected after acute/chronic infections and how this matters in the long run. In this review, we will focus on T cells and discuss the surface and molecular markers that are associated with T cell senescence. We will also look at the implications that senescent T cells could have on human health and diseases. Finally, we will discuss the benefits of having these markers for investigators and the future work that is needed to advance the field of T cell senescence markers.

## 1. Introduction

Cellular senescence and human aging research has been performed with much more attention in recent years. The aging population (alternatively named as the “grey tsunami”) together with the increased number of aged patients visiting healthcare institutions have been identified as potential upcoming burdens. The number of individuals aged 60 and above is expected to increase ~56% (900 million to 1400 million) from 2015–2030 and ~49% (1400 million to 2100 million) from 2030–2050. This dramatic increase in the number of older adults could have a huge impact on many economic and healthcare policies [1,2]. The impending “grey tsunami” is partly due to humans having a longer lifespan with the discovery of many medical interventions in the past few decades. Vaccines, drugs and antibiotics have saved many lives from life-threatening situations and infections, which were deemed incurable in the past [3,4,5]. This still greatly reduces the mortality rate of humans due to pathogens. However, having a longer life span brings up other issues, which are defined as age-related diseases such as dementia, rheumatoid arthritis, cancer, heart diseases and sarcopenia. These diseases have been associated with aging as they are more prevalent in the older population [6,7,8,9,10]. Although the discovery of vaccines enables us to train the immune system against harmful pathogens and it has prevented many deadly infections [11], hypo-responsiveness to vaccination is a barrier to further enhancement of healthy aging. The reduced efficacy of vaccines in the elderly could be due to the age-related changes in the immune system, also known as immunosenescence [12,13,14]. In the field of immunosenescence, a bulk of data exists on T cells and this is mainly explained by the array of markers identified to define the various subpopulations and functions [15,16]. Therefore, in this review, we will discuss and clarify the research on T cells, which are also the major subpopulation of lymphocytes in the human circulation. First, we will give a brief introduction to the immune system and the general concept of cellular senescence. Then we will discuss the markers that are commonly used in the field for T cells and their biological relevance. After understanding T cells senescence, we will identify its implication for human health and diseases. Finally, we will address future research, in terms of markers and phenotyping of T cells, with a focus on the T cells with an innate-like profile (Mucosal associated invariant T (MAIT), invariant natural killer T (iNKT) and γδ) as opposed to the classical adaptive T cells and other new players that are involved in cellular senescence.

## 2. Immune System, T Cells and Cellular Senescence

The immune system is made up of many different immune cell types, each with its own unique functions, to collectively protect the host against foreign pathogens [17,18]. T cells comprise around 7–24% of the immune cells and around ~70% of the lymphocytes in human blood. Classical T cells have the “memory” component, which allows them to respond faster in subsequent infection and are also long-lived [19,20]. They can be broadly classified into the Helper T cells (CD4) and Cytotoxic T Cells (CD8) [21,22]. CD4 T cells’ role in the immune system is to assist other immune cells in the different immunological processes [23]. To achieve this, CD4 T cells interacts with antigen presenting cells (APC) such as dendritic cells (DCs) with their surface receptors (CD27 to CD70 and CD28 to CD80/CD86) to get activated [24]. This allows T cells to proliferate extensively and secrete cytokines into the environment to aid the other immune cells. There are several subtypes of CD4 T cells based on the cytokines secreted, and this will facilitate the needs of various immunological processes such as B cell maturation and macrophage activation [25,26]. Cytotoxic CD8 T cells, as their name implies, kill virus-infected cells that present the viral antigen on major histocompatibility comples (MHC) Class 1 molecule. This is achieved by secreting molecules such as perforin and granzymes on the viral-infected cells, creating pores in the cell membrane and inducing apoptosis [27]. The ability of T cells to proliferate upon antigen stimulation is crucial as it dramatically increases the number of antigen-specific T cells to aid in resolving the infection, otherwise known as clonal expansion. After the resolution of the infection, these T cells undergo apoptosis during the contraction phase to return to the steady state [28]. However, as T cells replicate multiple times due to repeated stimulation with pathogens during a host’s lifetime, they further differentiate, lose their proliferation capacity and may reach the stage of replicative senescence [29,30]. The inability of T cells to proliferate is partly due to the erosion of telomeres and the loss of telomerase activity [31]. This phenomenon is analogous to the Hayflick Limit, which was established on fibroblasts, whereby Hayflick and co-workers found that the fibroblasts could no longer proliferate after ~50 passages in vitro [32]. Besides having an impaired proliferative capacity and shorter telomere length, senescent fibroblasts also adopt a pro-inflammatory profile, whereby they could secrete pro-inflammatory cytokines into the environment and cause tissue damage by chronic inflammation [33]. However, these features of senescence are established on the fibroblasts and classical T cells may shares similar features but the signals and pathways leading to those functional hallmarks may be different. Whether cellular senescence share common pathways across all immune cells and all mammalian cells still needs to be demonstrated.

## 3. Following T Cell Journey through Differentiation: Surface Markers as Guides

**Figure 1:**
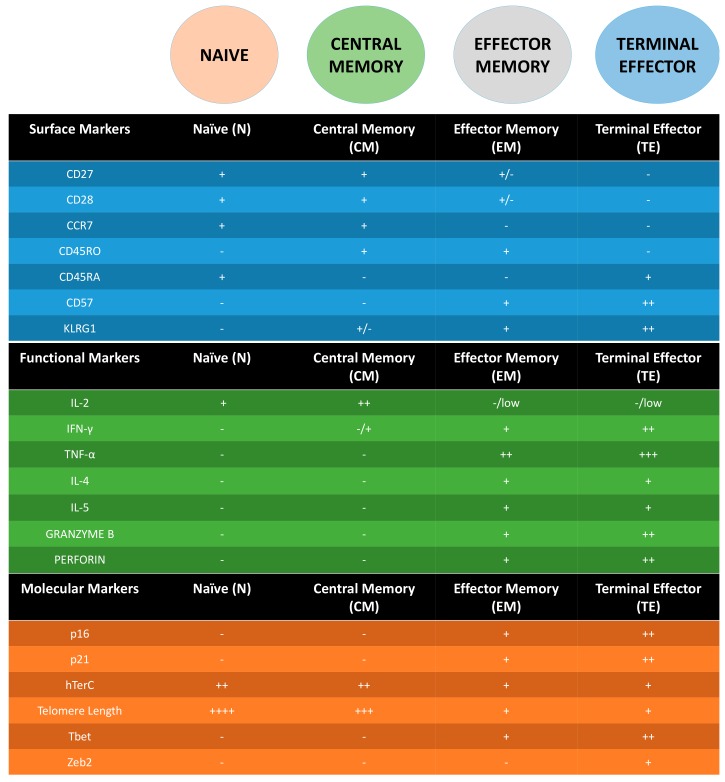
Summary table of surface, functional and molecular markers in the different stages for Adaptive T cells (CD4 and CD8). - = not expressed, + = expressed. Additional + = higher expression.

The study of T cell surface markers and functional capacity as it differentiates or “ages” is well studied and established [15,34,35,36,37,38]. Naïve (N) T cells express markers such as CD45RA and C-C chemokine receptor 7 (CCR7) [39], which allows them to home into the lymph nodes, along with CD27, CD28, which are co-stimulatory molecules that are used to interact with B cells and antigen presenting cells (APC) for the generation of immunoglobulin, long term maintenance and activation of T cells to produce cytokines [40]. As such, N T cells are defined as CD45RA+, CD45RO−, CD27+, CD28+ and CCR7+. Central Memory (CM) T cells (CD45RA−, CD45RO+, CD27+, CD28+ and CCR7+) are capable of producing high levels of interleukin-2 (IL-2) and interferon γ (IFNγ) (but not effector molecules such as tumor necrosis factor α TNFα, IL-4, IL-5 and cytotoxic molecules (e.g., perforin and granzymes)) [34,41]. Effector memory (EM) T cells (CD45RA−, CD45RO+, CD27−, CD28− and CCR7−) on the other hand have the capacity to produce high levels of effector molecules as mentioned above in general (although there are some differences between CD4 and CD8) but not IL-2. Lastly, terminal effector (TE) T cells (CD45RA+, CD45RO−, CD27−, CD28− and CCR7−) have limited proliferative capacity but tend to secrete a wider range of cytokines following activation [34]. T cells in a replicative senescence state, which are more prevalent in the EM and especially TE, do not express co-stimulatory molecules such as CD27 and CD28. Instead, they express markers such as Killer cell lectin-like receptor sub family G (KLRG-1) and CD57 [15,42]. KLRG-1 has an immunoreceptor tyrosine-based inhibitory motif (ITIM) and studies have shown that when KLRG-1 is prevented to ligate on T cells by blocking-antibodies, it enhanced the proliferation capacity through the increase of AKT, cyclin D, cyclin E and a decrease of cyclin inhibitor p27 [43,44]. CD57 on the other hand, is a glycoepitope and its expression is controlled by galactosylgalactosylxylosylprotein 3-β-glucuronosyltransferase 1 (B3GAT1). Even though the ligand for CD57 still remains unknown, studies have shown that the proliferation capacity of T cells expressing CD57 is severely impaired, suggesting CD57 to be the best marker of replicative senescence [45,46]. Therefore, using CD27, CD28, CD57, KLRG-1 and one of the CD45 isoforms (RA/RO) allows one to track the “age” of the circulating T cells. However, whether these markers could be applied to the innate-like T cells and whether a similar sequence of differentiation happens in tissues remains to be investigated (Figure 1).

## 4. Molecular Markers of Senescent T Cells

Following surface markers to understand T cell biology has been possible and eased by the use of technologies such as flow cytometry. However, while some markers have been biologically related to the process studied, some are just surrogate markers of T cell differentiation without knowledge of the molecular and signaling pathways responsible for the regulation of the marker or its function. The best example in the context of T cell senescence is CD57. Independent of surface markers, studies have demonstrated T cell regulation at the molecular level. Many of these molecular changes involve proteins associated with the telomeres or the cell cycle. With the loss of CD27 and CD28, molecules such as p16 and p21 that are involved in cell cycle regulation, are upregulated [47]. p16 is more associated with “stresses” that cause premature senescence and p21 that is directly induced by p53 and is more associated with senescence due to telomere damage [48,49,50]. Both regulate the cell cycle by inhibiting cyclin dependent kinase (CDK)4 and CDK6, which keeps retinoblastoma (RB) protein hypo-phosphorylated. This inhibits the cell cycle process of the transition from G1 to S phase, which leads to replicative senescence [51,52]. Another hallmark of senescent T cells is the shortening of telomeres. The shortening of telomeres is due to both the continuous replication of the T cells and a reduction in human telomerase RNA component (hTERC) expression, which is a factor for the telomerase activity [53]. The expression of hTERC has been associated with both CD27 and CD28 expression, suggesting that the loss of these surface markers could result in the reduction of the telomerase activity or vice versa (Figure 1). This reduced activity of telomerase has been associated with defective phosphorylation of Akt (Ser473) in CD27− CD28− subset [54]. However, by blocking KLRG-1 signalling pathway, another group showed that they were able to induce proliferation but not telomerase acitivity, even though Akt (Ser473) was upregulated [44]. This suggests that phosphorylation of Akt (Ser473) alone is not sufficient and might require other players such as ERK in order to restore telomerase activity. Another possibility for the reduction in telomerase activity could be intrinsic. Recently, a study has shown that the phosphorylation of p38 via AMPK and Tab1, inhibited telomerase activity and drove senescence of the T cell. This signaling was induced by low-nutrient sensing and DNA damage within the cell. Blockades of this pathway have been shown to restore the proliferative function of senescent T cells, which could be use in future therapy [55]. This concept has only been proven in T cells. Whether can we adopt a similar approach for other types of senescent cells (e.g., fibroblasts) remains to be investigated, as there could be underlying mechanisms that differentiate T cells and other cell types.

## 5. T Cells: Senescence Does Not Equate to Exhaustion

It is not surprising that investigators are often confused with the terms senescence and exhaustion of T cells. Senescence and exhausted T cells do have some similarity in certain aspects of functionality but they are not entirely the same [56]. Therefore, it is important to note the differences between senescence and exhaustion of T cells, as this will allow accurate interpretation of results and propose the right therapeutic approach to be used. First, the markers expressed by senescent T cells are markers such as CD57 and KLRG-1, which indicates replicative senescent [15]. On the other hand, the markers associated with exhaustion of T cells are programmed cell death 1 (PD-1), lymphocyte activation gene 3 (LAG-3), T cell immunoglobulin mucin 3 (TIM-3) and cytotoxic T lymphocyte-associated protein 4 (CTLA-4) [57]. Second, senescent T cells adopt a pro-inflammatory profile and are able to secrete high levels of pro-inflammatory cytokines with stimulation which is similar to the senescence associated secreting phenotype (SASP) that was established on fibroblasts [33]. The SASP concept has been established in non-immune cells but it remains to be proven in T cells. However, as SASP cells are unable to proliferate but can produce a higher range of pro-inflammatory molecules, it is likely that senescent T cells exhibit some aspects of SASP. This hypothesis may be true in view of the increasing diversity of cytokines produced in the sequence of T cell differentiation: N→CM→EM→TE→CD57/KLRG-1. On the contrary, exhausted T cells are unable to both proliferate and to secrete cytokine upon stimulation suggesting again that the two definitions refer to different cellular status. Third, senescent T cells are more prevalent in the highly-differentiated phenotypes (EM/TE) and resistant to apoptosis. Exhausted T cells on the other hand, are usually CM/EM T cells that have undergone repetitive or chronic stimulation [56]. They are programmed to undergo apoptosis as PD-1 pathway seems to strongly associate with survival [58].

Lastly, replicative senescent seems to be irreversible whereas exhaustion is reversible. Studies have shown that blockade of PD-1 ligation is able to recover the function of cytokine secretion in T cells [59,60]. “Reversing exhaustion” has been very successful in human clinical trials, raising the 5-year survival rate of different type of cancer patients in advanced cancer stages. Anti-PD1 (nivolumab) and anti-CTLA4 (Ipilimumab) are the two main candidates for checkpoint blockade immunotherapy currently [61,62,63,64,65]. However, many other checkpoint inhibitors such as anti-LAG3 and anti-TIM3 are also being explored to remove the “brakes” on the T cells, which will enable it to unleash its full functional capacity against cancer cells. As mentioned above, senescent T cells were recently shown to regain function by inhibiting p38 mitogen-activated protein kinase (MAPK) pathway [55]. Restoring function of senescent T cells is very relevant in the context of human aging while restoring the function of exhausted T cells is more relevant in a pathological context (e.g., cancer immunotherapy, infectious diseases). Having clarified the differences between senescent and exhausted T cells, the markers associated with each phenotype could be co-expressed on the surface of the T cells, which means they could be both senescent and exhausted. It is not clear, however, whether senescent T cells are more susceptible to exhaustion and vice-versa.

## 6. Implications of T Cells Senescence in Persistent Infections and Human Aging

Senescent T cells were shown to expand in patients with chronic and persistent infections such as cytomegalovirus (CMV), human immunodeficiency virus (HIV) along with an additive effect of chronological aging. CMV itself is asymptomatic in most of the hosts unless they are immune-compromised. However, the constant reactivation of the virus could have driven the accumulation of senescent T cells as the immune system tries to control virus reactivation [66,67]. HIV infection shares some similarities with the hallmarks of immunosenescence described above. It is of note that HIV is one of the situations where exhausted T cells are also present. HIV-infected patients usually exhibit high levels of inflammation molecules (IL-6, TNFα, C-reactive protein (CRP)), reduced vaccine efficacy and the expansion of senescent T cells. With the highly active antiretroviral therapy (HAART) therapy, the inflammatory status is reduced, suggesting that the constant replication of the virus could have driven inflammation [68]. In human aging, the accumulation of senescent T cells in the elderly is not surprising as the host have encountered a lifetime of infections, which could drive the differentiation of the T cells and ultimately reach the senescence stage [69]. The thymus, an organ where T cells develop and mature, involutes during aging and the main sites that are affected are the cortex and medulla though the mechanisms are unknown [70,71]. With a reduced in overall hematopoietic output that shifts towards a myeloid profile [72,73] and together with thymic involution, which results in decreased production of new naïve T cells [67], the T cell profile of the individual naturally shifts from being a “naive” profile (a profile that have less differentiated T cells: N and CM) to an “experienced” profile (a profile that have more differentiated T cells: EM and TE) as they age. Several interventions by systemic administration of cytokines have been able to partially restore the function of the thymus in the aging host as reviewed in [74,75]. However, none has been the magic bullet to completely restore the function and turn the thymus back to its “youthful days”. Thymic involution is happening during adulthood and is probably programmed to happen to adapt to the physiology of an older organism. It is conceivable to consider this as one of the mechanisms to save resources for other more important functions later in life. Obviously, naïve T cells are required in the first part of life where new antigens still need to be identified. Later in life this is probably less important compared to sustaining immunological memory. Altogether, there is a significant increase in the frequency of senescent T cells during aging. These cells in a resting state are able to secrete low levels of pro-inflammatory cytokines such as TNFα and IL-6 [33,47]. This will contribute to the low-grade systemic chronic inflammation in the elderly, which has been associated with many age-related diseases such as dementia, metabolic syndrome and heart diseases [76,77]. Next, senescent T cells also have a limited repertoire to antigens compared to naïve T cell diverse repertoire [78,79]. With an increase of senescent T cells and a decrease of naïve T cells in the elderly, this could diminish the “protection range and capacity” against pathogens in the elderly compared to the young. Therefore, the accumulation of senescent T cells is detrimental to the host but these beliefs might not hold true for other cell types such as fibroblast, whereby there are benefits of having senescent cells, such as wound healing [80,81].

## 7. Are the Markers Suitable for All T Cells?

The surface and molecular markers described above are well established for classical T cells. However, with the discovery of innate-like/non-classical T cells such as γδ T cells, mucosal associated invariant T (MAIT), invariant Natural Killer T cells (iNKT) and germline-encoded, mycolyl lipid–reactive (GEM) T cells in recent years, whether the markers expression on the surface of the adaptive counterpart has the same implication on these cell type remains to be investigated. Though these innate-like T cells might be less abundant in the periphery compared to the classical T cells, they contribute to the host defense by responding to other types of antigens and could proliferate extensively once activated [82,83]. They are also found to be more abundant in several tissues and contribute to the local immunosurveillence [84]. Recent work on the MAITs suggests that the classical T cell phenotype might not be applicable for the MAITs [85]. Though it is entirely possible that MAIT are not susceptible to cellular aging with chronological aging but there have also been no functional tests to indicate that the same markers and classification have to be used as compared to CD4 and CD8. For the γδ T cells, our work in 2014 suggested that, similar to MAIT, γδ T cells are either not susceptible to cellular aging or they simply do not follow the same rules as αβ T cells [86]. With further studies in the last two years, the data now seem to converge to the fact that Vδ2 (a subset of the γδ) does not follow the same markers as CD4 and CD8 (at least for CD27, CD28) in terms of cytokine production [87,88]. Eberl et al. also tested the biological relevance of ligating KLRG-1 on Vδ2 and it does not have the inhibitory effect as shown on CD4, CD8 and even natural killer (NK) cells [89]. This suggests that expression and implication of markers used are very different depending on the cell type. Surprisingly, Vδ1 (another subset of γδ) does follow the trend of CD8, an increase of “TE” phenotype in the periphery with CMV and an additive effect of human chronological aging [90]. A recent study by Davey et al. have also shown that CD27+CD45RA+ (“Naïve”) and CD27−CD45RA+ (TE) Vδ1 does have very different functional capacity and repertoire diversity, which is similar to CD8. However, this concept does not apply to Vδ2 [91]. These results collectively suggest that even within the same γδ T cell family, there is discrepancy in terms of marker expression and its implications. Besides surface markers comparison, another aspect that future research should address is the molecular and transcription factors that innate-like T cells express during the “differentiation” stages. Whether innate-like T cells exhibit similar molecular features as their adaptive counterpart such as p16, p21, shortened telomere length, reduced hTERC activity, being resistant to apoptosis during senescence and whether the “senescence programming” involves the high expression of Tbet and Zeb2 as shown in CD8 [92] remains to be investigated. Therefore, future work should look to elucidate relevant surface and molecular markers for these innate-like T cells, which will be useful for studies that are investigating this group of T cells in different diseases and conditions.

## 8. New Players in the Field of Senescence?

In addition to the markers mentioned above, recent studies have uncovered new players and their role in the field of cellular senescence. Mitochondria can be affected by cellular senescence in several aspects such as caveolin-1 deficiency, which has been shown to induce cellular senescence by affecting the functionality of the mitochondria [93]. The other aspect is mitochondrial DNA hypomethylation, which is a feature of induced senenscence in human fetal heart mesenchymal stem cells and can be induced by reactive oxidative species [94]. Lipid and nicotinamide (NAD) metabolism are also two other features of mitochondria that could be associated with cellular senescence as they are correlated with age-related diseases [95,96,97,98] Besides mitochondria, studies have investigated at the RNA level and assessed the role of non-coding RNA (i.e., micro RNAs, long noncoding RNAa and circular RNAs) with cellular senescence in aging organs/tissue. [99,100,101,102]. On the epigenetic level, CD4 and CD8 T cells have been shown to have an overall decrease in methylation as they progressed from Naïve to TE [103,104]. However, the epigenomic profile of innate and adaptive immune cells are distinctively different, which makes it hard to assess and compare, which could also mean that “road to senescence” is completely different [105]. At the DNA level, cGMP-AMP (cGAMP) synthase (cGAS), a cytosolic DNA sensor that associates itself with chromatin in high levels during DNA damage, is found to be essential for cellular senescence [106]. Collectively, these studies suggest that many aspects of cell biology are affected by cellular senescence; from surface marker protein expression down to the metabolism and DNA of the cell. However, whether these markers are associated and applicable to all cell types or whether there are unique pathways for specific cell type remains a question to be answered.

## 9. Conclusions

In conclusion, having markers of relevant biological functions in T cells such as CD57 and KLRG-1 (senescent markers) allows the community to understand the T cell profile in a particular condition with a simple flow cytometry experiment. This greatly increases the speed and feasibility of the analysis without the need to perform time-consuming, expensive and complex functional assays. Having said that, one must be careful in using these markers appropriately as the implication of marker expression between cell types could differ, as shown in Vδ2 T cells compared to CD4 and CD8. The discovery and use of more biologically relevant markers is definitely beneficial as flow cytometry enables investigators to perform much more complex phenotyping nowadays. With more parameters and options of reagents, users can input more parameters into the studies to elucidate the different phenotype of T cells and assess how different they are in various disease conditions. However, biomarkers are often only surrogate markers, often resulting from unknown mechanisms and more research is needed to understand the biological process behind the expression or repression of the candidate biomarkers. Therefore, markers are always useful, provided they have functional relevance.

## Abbreviations

APC	Antigen Presenting Cells
CD	Cluster of Differentiation
cGAS	cGMP-AMP Synthase
CM	Central Memory
CMV	Cytomegalovirus
CTLA-4	Cytotoxic T Lymphocyte-Associated Protein 4
DC	Dendritic Cells
DNA	Deoxyribonucleic Acid
EM	Effector Memory
GEM T	Germline-Encoded, Mycolyl T Cells
HIV	Human Immunodeficiency Virus
IFNγ	Interferon Gamma
iNKT	Invariant Natural Killer T Cells
IL	Interleukin
KLRG-1	Killer Lectin-Like Receptor Sub Family G Protein 1
*LAG3*	Lymphocyte Activation Gene 3
MAIT	Mucosal Associated Invariant T Cells
N	Naive
NAD	Nicotinamide
NK	Natural Killer
PD-1	Programmed Cell Death Protein 1
RNA	Ribonucleic Acid
RB	Retinoblastoma
SASP	Senescence Associated Secretory Phenotype
TEMRA	Terminal Effector Memory RA
TNFα	Tumor Necrosis Factor Alpha
